# Variations in quality of life in people with multimorbidity: A cross-sectional survey comparing MMQ1 and EQ-5D-5L

**DOI:** 10.1177/26335565251410275

**Published:** 2026-01-16

**Authors:** Kieran D. Sweeney, Dilek Coskun, Volkert Siersma, Lucy E. Stirland, Bruce Guthrie, Stewart W. Mercer, John B. Brodersen

**Affiliations:** 1 College of Medicine and Veterinary Medicine, Usher Institute, University of Edinburgh, Edinburgh, UK; 2 Centre of General Practice, Department of Public Health, Faculty of Health Sciences, University of Copenhagen, Copenhagen, Denmark; 3 Division of Psychiatry, Centre for Clinical Brain Sciences, University of Edinburgh, Edinburgh, UK; 4 Global Brain Health Institute, University of California San Francisco, San Francisco, USA; 5 Centre of Cancer and Organ Diseases, Copenhagen University Hospital, Copenhagen, Denmark; 6 Research Unit for General Practice, Department of Community Medicine, Faculty of Health Sciences, UiT The Arctic University of Norway, Tromsø, Norway

**Keywords:** multimorbidity, multiple long-term conditions, quality of life, patient reported outcome measures

## Abstract

**Background:**

The Multimorbidity Questionnaire (MMQ1) is a patient-reported outcome measure assessing quality of life (QoL) in people with multimorbidity. An English-language version was recently validated for use in the United Kingdom. This study examines: (1) Whether MMQ1 detects expected variations in QoL according to individual characteristics; (2) How MMQ1 compares with EQ-5D-5L in detecting such variations, and in discriminating between different levels of QoL.

**Methods:**

A postal survey was distributed to 2753 patients with multimorbidity. Relationships between MMQ1 and EQ-5D-5L with six independent variables (long-term condition count, mental-physical multimorbidity, deprivation, self-rated QoL, age and sex) were examined using linear regression analyses. Discriminative ability was assessed using Receiver Operating Characteristic curves and sample size calculations with respect to consecutive classes of self-rated QoL.

**Results:**

597 responses were received (22%). Respondents had a mean age of 69.5 years and 48% were men. Higher long-term condition count, the presence of mental-physical multimorbidity and increasing deprivation were associated with poorer QoL on both measures. In addition, three MMQ1 domains demonstrated age-related variations in QoL that were not detected using EQ-5D-5L. MMQ1 exhibited superior discriminative ability to EQ-5D-5L, especially in distinguishing between individuals with ‘Poor’ vs ‘Very Poor’ self-rated QoL, where EQ-5D-5L was particularly weak.

**Conclusion:**

MMQ1 detected expected variations in QoL according to individual characteristics, supporting known-groups validity. It was superior to EQ-5D-5L in its ability to detect age-related variations in QoL and to discriminate between different levels of self-rated QoL. MMQ1 has the potential to improve the measurement of QoL in people with multimorbidity.

## Introduction

There is a well-recognised need for patient reported outcome measures (PROMs) assessing quality of life (QoL) that are specifically tailored to people with multiple long-term conditions (also termed multimorbidity).^1–4^ In the absence of such bespoke PROMs, the measurement of QoL in this group has typically relied on generic utility measures such as EQ-5D-5L.^5,6^ Although EQ-5D-5L has well-established validity in many contexts, it was not designed for people with multimorbidity and has recognised psychometric limitations, including inadequate content validity.^4,7,8^ The reliance on generic QoL measures in multimorbidity research may be a contributory factor to the negative results of many multimorbidity intervention trials.^5,6^

To address this issue, the Multimorbidity Questionnaire (MMQ1) was recently developed and validated in Denmark.^9^ Using a conceptual framework of needs-based QoL – which is widely used in other single-disease QoL PROMs^10,11^ – MMQ1 was designed with and for people with multimorbidity. It consists of 37 items divided into six separate scales: Physical Ability, Concerns and Worries, Limitations in Daily Life, Social Life, Personal Finances and Self-image. The original Danish MMQ1 has now been translated into English and adapted and validated for use in the United Kingdom (UK), demonstrating good psychometric properties including high content validity and high correlation with EQ-5D-5L.^12^ By accounting for the complex and multifaceted impact of multimorbidity, MMQ1 aims to improve the measurement of QoL in people with multimorbidity compared with generic PROMs like EQ-5D-5L.^13^ However, the comparative performance of these two measures is not known.

This study analysed data from the MMQ1 UK validation survey in order to address two objectives:

1) To examine whether MMQ1 is able to detect expected variations in QoL across groups based on health and sociodemographic characteristics, thereby demonstrating known-groups validity.^14^
2) To compare MMQ1 with EQ-5D-5L in terms of (a) their respective ability to detect these variations across patient groups; and (b) their ability to discriminate between different levels of QoL as determined by a single-item global self-rating of QoL (*Very Good, Good, Acceptable, Poor, Very Poor*).

## Methods

The methods for the cross-sectional validation survey have been published in full previously.^12^ In summary, a postal survey including MMQ1 and EQ-5D-5L was sent to 2753 patients from eight primary care practices in the Lothian region of Scotland, UK. In participating practices, lists of registered patients were searched electronically to identify those on two or more long-term condition registers, or prescribed four or more repeat medications (a method used previously to identify patients with multimorbidity^15^). Multimorbidity was defined as the presence of two or more long-term conditions, and mental-physical multimorbidity was defined as the coexistence of at least one mental illness with at least one physical condition. A total of 11,860 potentially eligible patients were identified across eight practices, from which a random sample of 2800 patients (350 per practice) was selected for screening by a general practitioner (GP) based at each practice. Patients deemed inappropriate for survey inclusion by their GP, such as those with dementia or approaching end-of-life, were removed from the list (47 in total), with 2753 patients invited to complete the survey by post between November and December 2023. Data collection stopped at the end of January 2024. No reminders were sent and no reimbursement provided.

Survey packs included a cover letter, a participant information sheet, the questionnaire and a pre-paid return envelope. The questionnaire contents included MMQ1, EQ-5D-5L,^16^ two demographic items (age and sex), a single-item global self-rating of QoL, and a checklist of 17 common long-term conditions to assess multimorbidity,^17,18^ including free-text space to add conditions not listed (Supplemental Box 1). Each survey also included a code to identify the respondent’s GP practice. Practices were grouped according to whether they served areas of mainly low, mixed, or high deprivation, based on inspection of the distribution of Scottish Index of Multiple Deprivation for each practice’s registered population.^19^

## Analysis

Data was processed and analysed using R, version 4.4.1. EQ-5D-5L utility scores were calculated by converting the five item responses into a single summary value using a UK-specific value set, in accordance with guidance from the EuroQol group.^20^ Scores ranged from −0.157 (the weighted score for a response pattern of level 5 on all 5 dimensions) to 1 (indicating no problems on all 5 dimensions), with scores of less than 0 representing health states valued as worse than death. MMQ1 scale scores were calculated for each of the six domains within the measure: Physical Activity (scale 0-18), Concerns and Worries (scale 0-18), Limitations in Daily Life (scale 0-30), Social Life (scale 0-18), Personal Finances (scale 0-9) and Self-image (scale 0-18). For the purpose of sensitivity analysis, a summed MMQ1 total score (scale 0-111), comprising the sum of the six scales, was also calculated for each respondent. As such, there were eight outcome scales included in the analyses for this study: EQ-5D-5L, the six MMQ1 domains, and MMQ1 total.

For MMQ1 total and its domains, a *higher* score indicates *worse* needs-based QoL, meaning the scale is in the opposite direction to that of EQ-5D-5L utility. For both measures, sociodemographic characteristics of non-completers were compared with those of completers, with no significant differences found (Supplemental Table). In line with previous psychometric evaluation of MMQ1, analysis was then conducted for complete responses only, which comprised 96-99% of responses for EQ-5D-5L and each MMQ1 domain.

### Known-groups validity and association with health and sociodemographic characteristics

Based on existing literature,^21–25^ higher MMQ1 scores were expected to be associated with four known-group variables: increasing number of long-term conditions (LTCs), the presence of mental-physical multimorbidity, increased deprivation and poorer self-rated QoL. Significant differences were not expected for age or sex. To assess these hypotheses, firstly mean scores with 95% confidence intervals were calculated and plotted for all six independent variables (four known-group variables, plus age and sex). Secondly, simple linear regression models were fitted for all eight outcome scales, with each of the six independent variables as predictors (48 models in total). Effect sizes were reported using Cohen’s d for binary predictors (sex, mental-physical multimorbidity) and standardized β coefficients for continuous or ordinal predictors (age, LTC count, deprivation, self-rated QoL). Pearson correlation and R-squared were reported for each model. To aid interpretability, when reporting the output for EQ-5D-5L, the direction of effect was reversed to be consistent with MMQ1 (positive effect indicating worsening QoL). A p-value threshold of 0.01 was used to assess statistical significance, to account for multiple testing. If a relationship was demonstrated where it was not expected (i.e., for age or sex), this was explored using multiple linear regression to adjust for confounders.

### Discriminative ability

Discriminative ability was assessed in two ways. Firstly, Receiver Operating Characteristic (ROC) curves were computed for each pair of consecutive categories of self-rated QoL (i.e. *Very Good vs Good, Good vs Acceptable, Acceptable vs Poor,* and *Poor vs Very Poor*). The area under the ROC curve (AUC) was calculated for each threshold, with values of 1.0 representing perfect classification, values >0.7 indicating good discriminative ability, and values of 0.5 indicating poor discrimination equivalent to random chance. An average ROC curve assessed overall discriminating ability across all categories of self-rated QoL. Secondly, a sample size calculation was performed to determine the number of individuals needed to distinguish between the consecutive response thresholds of self-rated QoL in a t-test with 5% significance and 80% power. A lower number of individuals needed to distinguish between consecutive response groups indicates a more discriminative scale. For sensitivity analysis, the discriminative ability of the EQ-5D-5L level sum score (the sum of responses without conversion into utility values) was also examined using both the ROC-AUC and sample size methods.

To further visualise discriminative ability, mean scores for each category of self-rated QoL were plotted along with the distribution of scores on EQ-5D-5L and MMQ1 total. Global self-rated QoL was used as the reference for assessing discriminative ability in order to maintain consistency with the approach used in the evaluation of the original Danish MMQ1 by Bissenbakker et al,^9^ and because it provides a useful basis for future researchers to determine a minimally important difference.

## Results

The response rate was 597/2753 (22%). Table 1 gives the characteristics of survey respondents. The mean age of respondents was 69.5 years (range 21-97 years) and 48% were men. The proportion of patients living in areas of mixed deprivation was 58%, with 28% from areas of high deprivation, and 14% from areas of low deprivation. The mean number of LTCs was 3.7 (SD 1.94). 28% of respondents had mental-physical multimorbidity compared with 62% physical-only multimorbidity.

**Table 1.** Characteristics of respondents.

	Count (%)
	N= 597
Mean age, years (SD)	69.5 (12.8)
Age (years)	
<50	41 (7)
51-60	75 (13)
61-70	155 (26)
71-80	190 (32)
81-90	117 (20)
>90	14 (2)
Missing	5 (1)
Sex	
Male	285 (48)
Female	310 (52)
Missing	2 (0)
Deprivation group	
High	167 (28)
Mixed	347 (58)
Low	83 (14)
Self-reported LTC count	
0-1^a^	59 (10)
2-3	236 (40)
4-6	197 (33)
6+	105 (18)
Multimorbidity type	
Mental-physical	165 (28)
Physical only	373 (62)
Missing^a^	59 (10)

LTC = Long-term condition.

^a^Patients who self-reported 0-1 conditions were included in the analysis on the basis of their GP electronic record.

### Known-groups validity and association with health and sociodemographic characteristics

**Figure 1.**
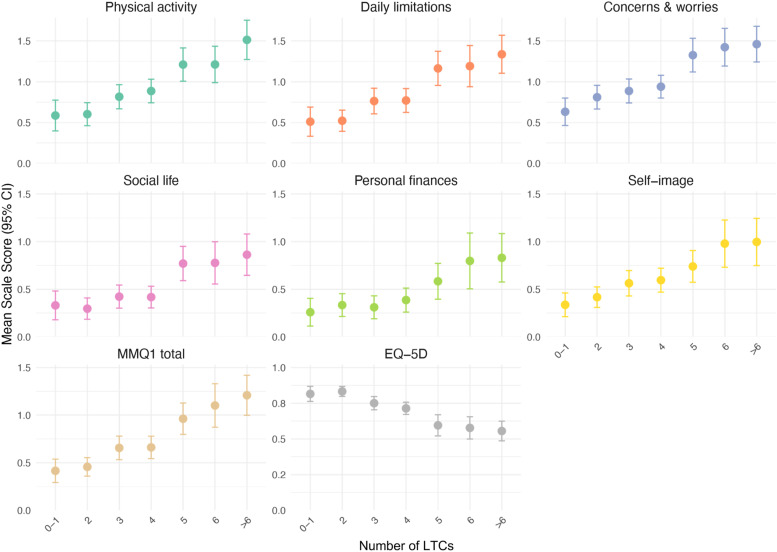
Relationship between mean outcome scores and number of self-reported long-term conditions (LTC). Note: Scale direction for EQ-5D-5L is opposite to that of MMQ1 and its subscales. Higher scores for MMQ1 indicate worse qulity of life, while higher scores on EQ-5D-5L indicate better quality of life. All MMQ scales have been standardised by diving mean score by number of items.

Figure 1 shows the variations in mean scores with increasing LTC count for each scale, demonstrating a clear dose-response relationship between increasing number of LTCs and worsening QoL. Expected differences were also demonstrated in figures for mental-physical multimorbidity, deprivation and self-rated QoL (Supplemental Figures 1-3).

Table 2 gives the results of 48 simple linear regression models, comparing the associations between the six independent variables and the eight outcome scales. As hypothesized, statistically significant (p < 0.01) associations were demonstrated in the expected directions for all four known-group variables (LTC count, mental-physical multimorbidity, deprivation and self-rate QoL) in all six domains of MMQ1, as well as MMQ1 total and EQ-5D-5L. Number of LTCs showed consistent small to moderate associations with all eight outcome scales (β = 0.23-0.36), with stronger effects for Physical Activity (β = 0.34), MMQ Total (β = 0.36) and EQ-5D-5L (β = 0.35). Approximately 13% of the variance in both MMQ1 Total and EQ-5D-5L scores was explained by LTC count. Having mental-physical multimorbidity (vs. physical only) was associated with medium to large effects across all scales (d = 0.49-0.87) with the strongest impacts on Self-image (d = 0.87) and Personal Finances (d = 0.76). Deprivation showed small but statistically significant associations across all eight scales (β = 0.13-0.17). Finally, global self-rating of QoL demonstrated strong associations with all MMQ1 domains (β = 0.55-0.79, R^2 =^ 0.31-0.62). In general, findings were highly consistent between MMQ1 and EQ-5D-5L, with MMQ1 total demonstrating slightly stronger associations (based on effect sizes, correlations and R^2^) for all three known-group variables (Table 2).

**Table 2.** Effect sizes, p-value, correlation (r) and R^2^ output from 48 simple linear regression models.

Predictor Variable	Physical Activity	Concerns & Worries	Daily Limitations	Social Life	Personal Finances	Self-Image	MMQ Total	EQ-5D-5L
LTC count	0.338 [0.26, 0.415] p < 0.001 r = 0.336 R^2^ = 0.1130	0.304 [0.226, 0.382] p < 0.001 r = 0.304 R^2^ = 0.0927	0.316 [0.237, 0.395] p < 0.001 r = 0.314 R^2^ = 0.0987	0.267 [0.188, 0.347] p < 0.001 r = 0.267 R^2^ = 0.0713	0.231 [0.151, 0.31] p < 0.001 r = 0.230 R^2^ = 0.0529	0.280 [0.201, 0.359] p < 0.001 r = 0.280 R^2^ = 0.0782	0.357 [0.276, 0.438] p < 0.001 r = 0.357 R^2^ = 0.1275	0.352 [−0.428, −0.276] p < 0.001 r = 0.354 R^2^ = 0.1250
Mental-physical multimorbidity (ref = physical-only)	0.486 [0.3, 0.672] p < 0.001 r = 0.213 R^2^ = 0.0454	0.719 [0.532, 0.906] p < 0.001 r = 0.308 R^2^ = 0.0947	0.524 [0.337, 0.711] p < 0.001 r = 0.228 R^2^ = 0.0522	0.711 [0.523, 0.9] p < 0.001 r = 0.303 R^2^ = 0.0916	0.755 [0.568, 0.943] p < 0.001 r = 0.320 R^2^ = 0.1024	0.873 [0.681, 1.065] p < 0.001 r = 0.362 R^2^ = 0.1309	0.756 [0.556, 0.956] p < 0.001 r = 0.320 R^2^ = 0.1021	0.723 [−0.911, −0.535] p < 0.001 r = 0.307 R^2^ = 0.0943
Deprivation level	0.168 [0.087, 0.25] p < 0.001 r = 0.168 R^2^ = 0.0281	0.166 [0.085, 0.247] p < 0.001 r = 0.165 R^2^ = 0.0273	0.169 [0.087, 0.25] p < 0.001 r = 0.168 R^2^ = 0.0284	0.14 [0.059, 0.222] p < 0.001 r = 0.140 R^2^ = 0.0197	0.126 [0.045, 0.207] p = 0.0024 r = 0.126 R^2^ = 0.0158	0.132 [0.051, 0.213] p = 0.0015 r = 0.133 R^2^ = 0.0176	0.174 [0.088, 0.26] p < 0.001 r = 0.174 R^2^ = 0.0302	0.159 [−0.239, −0.078] p < 0.001 r = 0.159 R^2^ = 0.0252
Global self-rated QoL	−0.706 [−0.763, −0.649] p < 0.001 r = −0.720 R^2^ = 0.5190	−0.721 [−0.778, −0.663] p < 0.001 r = −0.725 R^2^ = 0.5258	−0.712 [−0.769, −0.655] p < 0.001 r = −0.725 R^2^ = 0.5251	−0.676 [−0.735, −0.617] p < 0.001 r = −0.689 R^2^ = 0.4744	−0.547 [−0.616, −0.479] p < 0.001 r = −0.553 R^2^ = 0.3062	−0.685 [−0.746, −0.625] p < 0.001 r = −0.689 R^2^ = 0.4743	−0.789 [−0.844, −0.735] p < 0.001 r = −0.788 R^2^ = 0.6213	−0.692 [0.633, 0.751] p < 0.001 r = −0.696 R^2^ = 0.4850
Age	0.025 [−0.058, 0.108] p = 0.5582 r = 0.025 R^2^ = 0.0006	−0.194 [−0.275, −0.113] p < 0.001 r = −0.194 R^2^ = 0.0376	0.023 [−0.06, 0.106] p = 0.5842 r = 0.023 R^2^ = 0.0005	−0.105 [−0.186, −0.023] p = 0.0118 r = −0.105 R^2^ = 0.0111	−0.287 [−0.365, −0.209] p < 0.001 r = −0.289 R^2^ = 0.0834	−0.282 [−0.362, −0.203] p < 0.001 r = −0.281 R^2^ = 0.0788	−0.096 [−0.182, −0.009] p = 0.0302 r = −0.096 R^2^ = 0.0092	−0.091 [0.009, 0.172] p = 0.0299 r = −0.091 R^2^ = 0.0082
Sex (ref = male)	−0.103 [−0.268, 0.062] p = 0.2192 r = −0.052 R^2^ = 0.0027	−0.160 [−0.324, 0.005] p = 0.0562 r = −0.080 R^2^ = 0.0064	−0.061 [−0.225, 0.104] p = 0.4704 r = −0.030 R^2^ = 0.0009	−0.086 [−0.251, 0.078] p = 0.3021 r = −0.043 R^2^ = 0.0019	−0.032 [−0.196, 0.131] p = 0.6959 r = −0.016 R^2^ = 0.0003	−0.139 [−0.303, 0.026] p = 0.0987 r = −0.069 R^2^ = 0.0048	−0.068 [−0.242, 0.105] p = 0.4412 r = −0.034 R^2^ = 0.0012	−0.103 [−0.06, 0.267] p = 0.2151 r = −0.052 R^2^ = 0.0027

Each cell contains the model effect size with 95% confidence interval, p-value, correlation coefficient (r) and R^2^. Effect sizes: Cohen’s d for binary predictors (sex, mental-physical multimorbidity), Standardized β for continuous predictors (age, LTC count, deprivation). LTC = long-term condition.

Direction for effect for EQ-5D-5L corrected to match MMQ1.

Green predictor variables on the left are those where an associated was expected, whereas no differences were expected for blue predictor variables.

No significant differences in QoL were expected based on age or sex. While this hypothesis was supported for sex, age showed unexpected associations across three MMQ1 domains, with small negative effects for Concerns and Worries (β = −0.19, p < 0.001), Personal Finances (β = −0.29, p < 0.001), and Self-image (β = −0.28, p < 0.001). This relationship was further examined using multiple linear regression while adjusting for confounders (LTC count, mental-physical multimorbidity and deprivation). In the adjusted model (Table 3), these age-related associations remained statistically significant, with small effect sizes (Standardized β 0.13-0.22). The direction of these effects suggests that younger people with multimorbidity experience poorer quality of life in these three domains than older people. This relationship is visualised in Supplemental Figure 4, which shows negative correlations with age for these three MMQ1 domains (Concerns and Worries, Self-image, and Personal Finances). EQ-5D-5L, MMQ1 total and the Social Life domain of MMQ1 all demonstrated non-significant trivial effects in the same direction in both the adjusted and unadjusted models, while the remaining MMQ1 domains (Physical Activity and Daily Limitations) demonstrated non-significant trivial effects in the opposite direction (older age associated with worsening QoL).

**Table 3.** Multiple linear regression model output showing adjusted effects for age across eight outcome scales.

Predictor variable	Physical activity	Concerns & worries	Daily limitations	Social life	Personal finances	Self-image	MMQ total	EQ-5D-5L
Age	0.076 [−0.006, 0.158]	−0.131* [−0.211, −0.051]	0.087 [0.005, 0.169]	−0.026 [−0.106, 0.054]	−0.219* [−0.297, −0.141]	−0.207* [−0.285, −0.129]	−0.021 [−0.103, 0.061]	0.022 [−0.056, 0.1]
LTC count	0.287* [0.201, 0.373]	0.233* [0.151, 0.315]	0.249* [0.163, 0.335]	0.175* [0.091, 0.259]	0.152* [0.07, 0.234]	0.19* [0.108, 0.272]	0.274* [0.188, 0.36]	−0.272* [−0.354, −0.19]
Deprivation	0.178* [0.102, 0.254]	0.163* [0.089, 0.237]	0.176* [0.1, 0.252]	0.155* [0.079, 0.231]	0.126* [0.052, 0.2]	0.141* [0.068, 0.214]	0.179* [0.101, 0.257]	−0.168* [−0.242, −0.094]
Mental-physical multimorbidity (ref = physical-only)	0.277* [0.075, 0.479]	0.408* [0.214, 0.602]	0.363* [0.161, 0.565]	0.526* [0.326, 0.726]	0.44* [0.246, 0.634]	0.509* [0.315, 0.703]	0.47* [0.266, 0.674]	−0.432* [−0.628, −0.236]
Model R^2^	0.154	0.181	0.147	0.143	0.178	0.212	0.199	0.184

All predictors included simultaneously. Values show standardized β coefficients from the adjusted model, with 95% confidence intervals in brackets. * p<0.01.

LTC = long-term condition.

### Discriminative ability

Table 4 presents the results of the assessment of discriminative ability using both the ROC-AUC and sample size methods. ROC plots are provided in Supplemental Figure 5. Overall, MMQ1 demonstrated superior discriminative ability to EQ-5D-5L. In particular, MMQ1 Total demonstrated the best discriminative ability of all nine scales tested, with the highest value for mean AUC (0.75), and the smallest mean sample size result (40). Four MMQ1 domain scales (Physical Activity, Daily Limitations, Concerns & Worries, and Social Life) also showed strong discriminative ability, with mean AUC values ranging from 0.71 to 0.74 and mean sample sizes results ranging from 47 to 62. EQ-5D-5L demonstrated weaker discriminative ability (mean AUC = 0.68, mean sample size = 1927), as did the Personal Finances (mean AUC = 0.62, mean sample size = 154) and Self-image (mean AUC = 0.67, mean sample size = 146) domains of MMQ1. In sensitivity analysis, EQ-5D-5L level sum score performed similarly to the utility score used in the primary analysis (mean AUC = 0.67, mean sample size = 469).

**Table 4.** Discriminative ability examined using i) area under the curve (AUC) for receiver operative characteristic curves, and ii) sample size (SS) calculations, for each pair of consecutive categories of self-rated QoL.

Scale	Very Good vs Good	Good vs Acceptable	Acceptable vs Poor	Poor vs Very Poor	Mean performance	Rank
MMQ1: Physical activity	AUC = 0.632 SS = 62	AUC = 0.798 SS = 28	AUC = 0.714 SS = 54	AUC = 0.684 SS = 78	AUC = 0.707 SS = 56	AUC: #5 SS: #4
MMQ1: Concerns & worries	AUC = 0.643 SS = 54	AUC = 0.758 SS = 32	AUC = 0.732 SS = 46	AUC = 0.724 SS = 56	AUC = 0.714 SS = 47	AUC: #4 SS: #2
MMQ1: Daily limitations	AUC = 0.695 SS = 72	AUC = 0.787 SS = 32	AUC = 0.765 SS = 34	AUC = 0.718 SS = 70	AUC = 0.741 SS = 52	AUC: #2 SS: #3
MMQ1: Social life	AUC = 0.623 SS = 112	AUC = 0.773 SS = 48	AUC = 0.739 SS = 50	AUC = 0.782 SS = 36	AUC = 0.729 SS = 62	AUC: #3 SS: #5
MMQ1: Personal finances	AUC = 0.483 SS = 282	AUC = 0.670 SS = 104	AUC = 0.690 SS = 82	AUC = 0.645 SS = 148	AUC = 0.622 SS = 154	AUC: #9 SS: #7
MMQ1: Self-image	AUC = 0.587 SS = 92	AUC = 0.772 SS = 40	AUC = 0.693 SS = 68	AUC = 0.619 SS = 382	AUC = 0.668 SS = 146	AUC: #8 SS: #6
MMQ1 total score	AUC = 0.724 SS = 48	AUC = 0.814 SS = 24	AUC = 0.775 SS = 24	AUC = 0.703 SS = 62	AUC = 0.754 SS = 40	AUC: #1 SS: #1
EQ-5D-5L utility score	AUC = 0.656 SS = 74	AUC = 0.772 SS = 44	AUC = 0.775 SS = 34	AUC = 0.515 SS = 7554	AUC = 0.680 SS = 1927	AUC: #7 SS: #9
EQ-5D level sum score	AUC = 0.635 SS = 64	AUC = 0.781 SS = 28	AUC = 0.773 SS = 34	AUC = 0.562 SS = 1750	AUC = 0.688 SS = 469	AUC: #6 SS: #8

AUC: Area under the curve from the receiver operative characteristic (ROC) curves for consecutive categories of self-rated QoL. ROC curves are shown in Supplemental Figure 5. Higher values indicate better discriminate ability, with values above 0.7 considered good (1.0 = perfect classification, 0.5 = equivalent to random guessing).

SS: Sample size needed to discriminate between consecutive categories of self-rate QoL with 5% significance and 80% power. Lower values indicate more discriminating scales.

Rank gives the positional order (from 1-9) of each scale based on AUC and SS mean performance, in order to aid interpretation. Note that for AUC, higher values are better, whereas for SS, lower values are better.

At the level of the individual thresholds (pairs of consecutive categories of self-rated QoL), MMQ1 showed particularly strong discriminative ability at the *Good vs Acceptable* threshold (MMQ1 Total: AUC = 0.81, sample size = 24). Five of the six MMQ1 domains had an AUC >0.75, and sample size <50 at this threshold. EQ-5D-5L showed particularly weak discriminative ability at the *Poor vs Very Poor* threshold (AUC = 0.52, sample size = 7554). EQ-5D-5L was most discriminating at the *Acceptable vs Poor* threshold, with an AUC similar to that of MMQ1 Total (MMQ1 Total AUC = 0.78, EQ-5D-5L AUC = 0.77). However, all of the MMQ1 domain scales also demonstrated good discriminative ability at this threshold (AUC range = 0.69 to 0.77, sample size range = 34 to 82).

**Figure 2.**
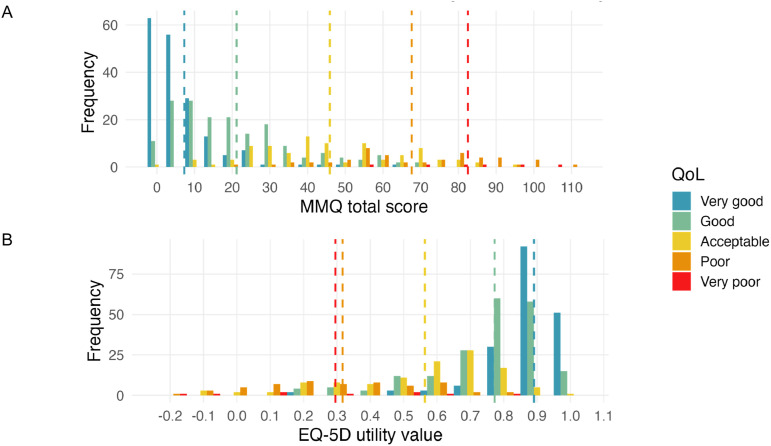
Mean scores for five QoL categories across the distribution of responses for (A) MMQ1 and (B) EQ-5D-5L. Distribution of MMQ total and EQ-5D-5L scores, grouped by overall QoL rating (colours). Vertical dashed lines indicate the mean for each group. Poorly separated lines indicate weak discriminative ability. Note: Scale direction for EQ-5D-5L is opposite to that of MMQ1 and its scales. Higher scores for MMQ1 indicate worse quality of life, while higher scores on EQ-5D-5L indicate better quality of life.

A further visual comparison of discriminative ability between MMQ1 and EQ-5D-5L is given in Figure 2, which shows the distribution of MMQ1 total and EQ-5D-5L utility scores for all 597 respondents, along with the position of the mean values for the five categories of self-rated QoL. While the mean values for MMQ1 total in these five groups are approximately evenly distributed along the scale, the five mean values for EQ-5D-5L are poorly separated, particularly between *Poor* and *Very Poor*, emphasising the weak discriminative ability of EQ-5D-5L at this threshold.

## Discussion

In this analysis of data from a cross-sectional validation survey in Scotland, MMQ1 demonstrated expected associations with number of long-term conditions, presence of mental-physical multimorbidity, deprivation and global self-rated QoL, thereby supporting the known-groups validity of this novel instrument.^14^ The strongest association was for self-rated QoL, providing evidence of robust convergent validity for MMQ1. Across the other known-group variables, effect sizes were highest for mental-physical multimorbidity, indicating the substantial impact of mental health conditions on the burden of multimorbidity, particularly on the Self-image domain of QoL. More modest yet consistent effect sizes were seen for LTC count, with results indicating that for every one standard deviation increase in the number of LTCs, there was a 0.23-0.36 standard deviation increase in scale scores. Smaller effect sizes were seen with deprivation but given that this was measured using only three broad categories (low, mixed, high), these effects are still noteworthy, suggesting that MMQ is sensitive to socioeconomic gradients even when measured coarsely.

In addition, three MMQ1 scales (Concerns and Worries, Self-image, and Personal Finances) demonstrated unexpected and small but statistically significant age-related associations, suggesting that the negative impact of multimorbidity on these domains of QoL is greater in younger adults. These age-related differences were not detected using EQ-5D-5L, suggesting that the six-scale structure and needs-based conceptual framework of MMQ1 leads to greater sensitivity in capturing variations in QoL in people with multimorbidity than EQ-5D-5L.

Assessment of discriminative ability further supports this conclusion, finding that MMQ1 was generally superior to EQ-5D-5L in distinguishing between consecutive categories of self-rated QoL, and in particular between *Poor* and *Very Poor*, where EQ-5D-5L demonstrated especially weak discriminative ability. The certainty of these findings is strengthened by the concordance of results from the ROC-AUC and sample size methods.

The known-groups validity assessment in this study was underpinned by existing literature demonstrating a consistent association between multimorbidity and poorer quality of life, and in particular, associations between lower QoL and increasing number of LTCs, the presence of mental health conditions and increased deprivation are well established.^21–25^ Indeed, Jørgensen et al. recently demonstrated an almost linear dose-response relationship between number of conditions and MMQ1 scale scores using the original Danish version of this measure.^26^ The relationship between age and QoL found in this study, while unexpected in the context of the known-group assessment, is supported by other studies which have reported a greater impact on QoL from multimorbidity in younger adults.^21–25,27,28^

### Underlying differences between MMQ1 and EQ-5D-5L

There are several key differences between MMQ1 and EQ-5D-5L which underpin the comparative findings of this study. Firstly, MMQ1 has 37 items, compared with just five in EQ-5D-5L, the former allowing assessment of QoL across a wider spectrum and with greater granularity. While the five items in EQ-5D-5L represent distinct dimensions of QoL, this instrument is typically used to provide a single utility score or ‘profile’. By comparison, the 37 items in MMQ1 constitute six separate validated scales, providing six separate scores. This structure enhances MMQ1’s ability to detect subtle variations across different domains of QoL, which could be missed when using only one sum score – something evident in the age-related differences observed in this study.

Secondly, the conceptual and measurement frameworks of the two PROMs differ. MMQ1 is based on a reflective model, where the items within a scale reflect an underlying construct, whereas EQ-5D-5L is a composite measure, with its five dimensions combining to define the health state. This means that the measures have different theoretical relationships between observed items and the underlying construct. Furthermore, EQ-5D-5L measures health-related QoL, while MMQ1 measures needs-based QoL. The latter is a theory-driven, broader conceptualisation of QoL, which has an explicitly temporal, adaptive quality, and is rooted in the idea that QoL is determined by the extent to which an individual is able to meet their own needs and goals – something that changes over the life course.^27^ This conceptual difference is particularly relevant to the fact that MMQ1 detected age-related variations where EQ-5D-5L did not. The fact that younger people with multimorbidity reported poorer QoL in particular domains of MMQ1 (notably those reflecting more psychosocial than physical dimensions of QoL), may be due to differences in expectations or perceived norms among younger adults, something that is not accounted for in the narrower, health-related conceptual framework of EQ-5D-5L. While a detailed examination of the pattern and implications of these age-related variations in QoL was beyond the scope of this paper, these findings highlight an important avenue for future research.

A third area of difference is in design and purpose. MMQ1 is a bespoke multimorbidity PROM, meaning that its items have been specifically tailored to address aspects of QoL deemed important by people with multimorbidity, and designed for use in multimorbidity intervention trials and observational studies. By contrast, EQ-5D-5L is a generic PROM (designed to be applicable to a range of health conditions and contexts), and a multi-attribute utility instrument (meaning that it can used to estimate utilities based on population preferences to calculate quality adjusted life years). As such, the two measures serve somewhat different purposes, and MMQ1 could not replace EQ-5D-5L in contexts where utility estimates or economic analyses are required. However, the head-to-head comparison provided by this study would be of interest to researchers who intend to measure quality of life as an outcome in people with multimorbidity, where previously only generic measures were available.

These differences between EQ-5D-5L and MMQ1 likely underpin their comparative performance in this study, including the marked superiority of MMQ1 in discriminating between those with *Poor vs Very Poor* global self-rated QoL rating. This particular finding suggests that trials targeting individuals with ‘complex multimorbidity’ (such as that involving both mental and physical conditions^29^) may be especially better served using MMQ1 over EQ-5D-5L, given that lower baseline QoL would be expected, and therefore any improvement may often be in the *Poor* vs *Very Poor* QoL range. This finding also raises the hypothetical question of whether previous multimorbidity trials, such as the 3D study,^6^ might have demonstrated improvements in QoL had MMQ1 been available to use as an outcome measure. That said, the choice of measurement instruments for any given context also depends on several wider factors including the design and purpose of the study, the mode of administration and need for economic analysis.

### Strengths and limitations

In demonstrating known-groups validity, this study makes an important contribution to the psychometric validation of MMQ1. The use of regression analyses provided a robust assessment of known-group validity. The direct comparison between MMQ1 and EQ-5D-5L in terms of (a) variations across sociodemographic groups, and (b) discriminative ability, also provides insight into the comparative performance of these two instruments. Limitations include that the survey response rate in this study was relatively low (22%) and representativeness could not be assessed, although this would apply equally to MMQ1 and EQ-5D-5L. The paper-based mode of administration, which requires a certain level of function from respondents, may have introduced selection bias. This limitation would also apply to both measures, although the comparison does not account for the fact that response rates (and representativeness) may have differed had the two measures been distributed independently, given that EQ-5D-5L is considerably shorter and therefore has a lower respondent burden. The survey pack did not include the EQ-Visual Analogue Scale and the comparison focussed on the composite profile score of EQ-5D-5L, rather than individual dimensions. While this is consistent with how the instrument is most commonly reported, it does mean that there were important conceptual differences between the instruments being compared, as discussed above.

The analyses were intended to compare the psychometric properties of MMQ1 and EQ-5D-5L, rather than to make generalisable conclusions regarding QoL in people with multimorbidity. That said, the findings of this study are consistent with existing literature. For the purpose of comparison with EQ-5D-5L, this study included MMQ1 total scores as a crude aggregate of its six scales, but it is important to note that the intended use of MMQ1 is as six separate scales, used concurrently. In the context of a trial, this structure entails a more complicated approach to sample size calculation. However, the feasibility of using MMQ1 in a trial has been successfully demonstrated by the recent MM600 trial in Denmark, which used MMQ1 as its primary outcome measure and obtained a response rate of 22% from a study population of almost 160,000 patients.^26,30^

Finally, this was a cross-sectional study, assessing discrimination and known-group validity based on a single measurement timepoint. Repeated, longitudinal assessment would enable more robust psychometric evaluation of MMQ1 and comparison with EQ-5D-5L, in particular of responsiveness, which is arguably the single most important measurement property for intervention studies.

## Conclusion

This study supports the use of MMQ1 as a robust measure of QoL in people with multimorbidity. In each of its six scales, it effectively demonstrated expected associations with number of LTCs, presence of mental-physical multimorbidity, deprivation and self-rated QoL. Moreover, it demonstrated superior performance to EQ-5D-5L in detecting age-related differences in QoL, and in discriminating between different levels of self-rated QoL. As such it has the potential to improve the measurement of QoL in people with multimorbidity, including clinical trials, prognostic studies and quality assessment. Future research examining the responsiveness of MMQ1 to changes in health status over time, such as post-interventions, would further substantiate its validity.

## Supplemental Material

**Supplemental Material -** Variations in quality of life in people with multimorbidity: A cross-sectional survey comparing MMQ1 and EQ-5D-5L Supplemental Material for Variations in quality of life in people with multimorbidity: A cross-sectional survey comparing MMQ1 and EQ-5D-5L by Kieran D. Sweeney, Dilek Coskun, Volkert Siersma, Lucy E. Stirland, Bruce Guthrie, Stewart W. Mercer, John Brodersen in Journal of Multimorbidity and Comorbidity.

## Data Availability

Consent was not obtained for making survey data publicly available.*
