# Supplementary material for: Variations in quality of life in people with multimorbidity: A cross-sectional survey comparing MMQ1 and EQ-5D-5L
Source: J Multimorb Comorb. 2026 Jan 16;16:26335565251410275. doi: 10.1177/26335565251410275 (PMC12811566; doi:10.1177/26335565251410275)
Supplement: Supplemental Material - Variations in quality of life in people with multimorbidity: A cross-sectional survey comparing MMQ1 and EQ-5D-5L [file sj-pdf-1-cob-10.1177_26335565251410275.pdf]

Supplementary Material

Supplementary Box 1. Self-reported checklist of common long-term conditions used to measure multimorbidity

About my health

Has a doctor or healthcare professional <u>ever told you</u> that you have had one of the following? (please tick as many boxes as apply to you)

High blood pressure ☐	Diabetes ☐	Angina/heart attack ☐	Heart Failure ☐
Stroke/TIA ☐	Arthritis ☐	Back problems ☐	COPD ☐
Eczema/psoriasis ☐	Asthma ☐	Thyroid problems ☐	Migraine ☐
Anxiety/depression ☐	Kidney disease ☐	Liver disease ☐	Cancer ☐

Irritable bowel syndrome ☐ Other (please state ___________________________) ☐

Supplementary Figure 1. Mean outcome scores for patients across three levels of deprivation

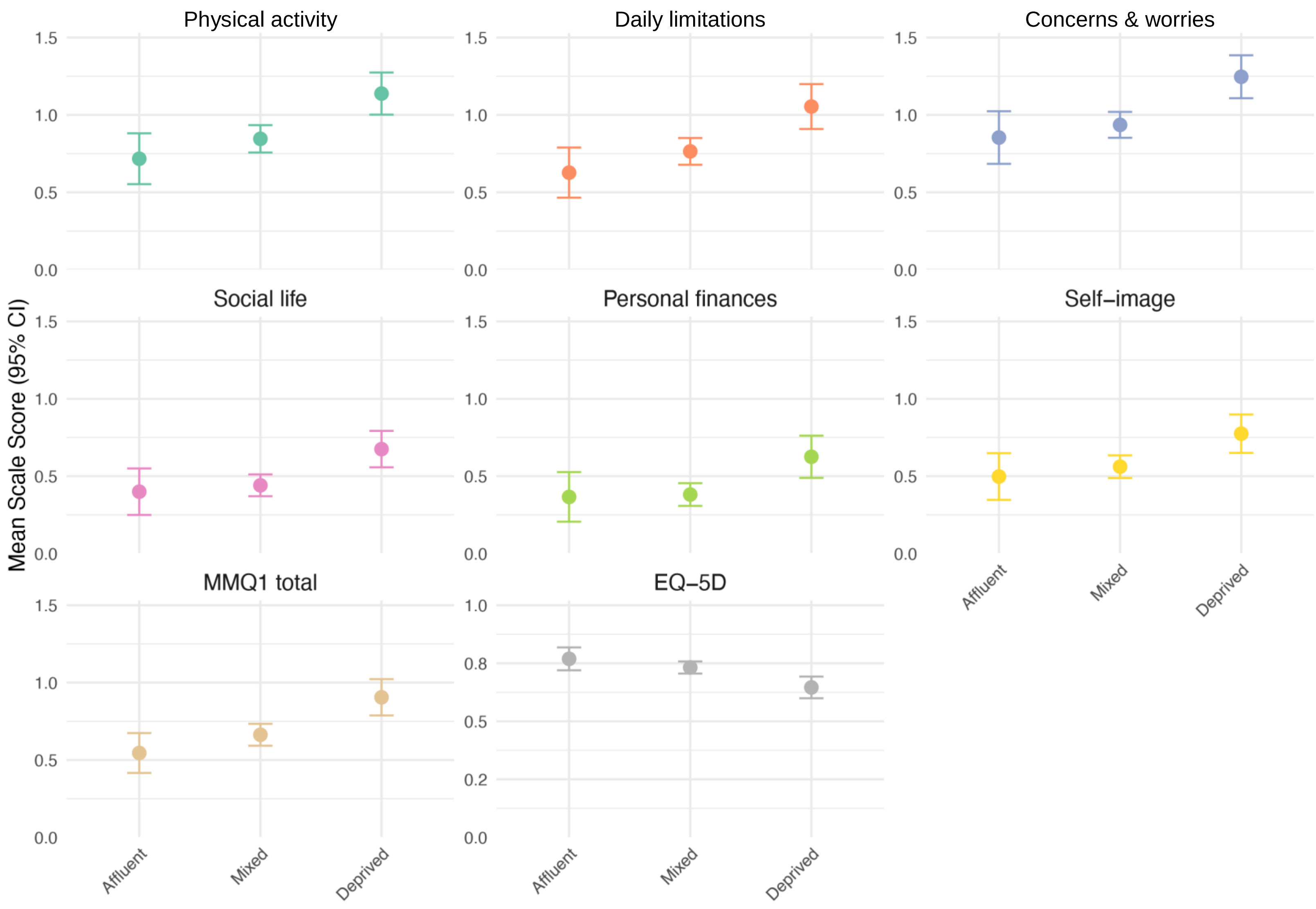

Note: Scale direction for EQ-5D-5L is opposite to that of MMQ1 and its subscales. Higher scores for MMQ1 indicate worse qulity of life, while higher scores on EQ-5D-5L indicate better quality of life. All MMQ scales have been standardised by diving mean score by number of items.

Supplementary

Figure 2. Mean outcome scores for those with mental-physical multimorbidity compared with physical-only

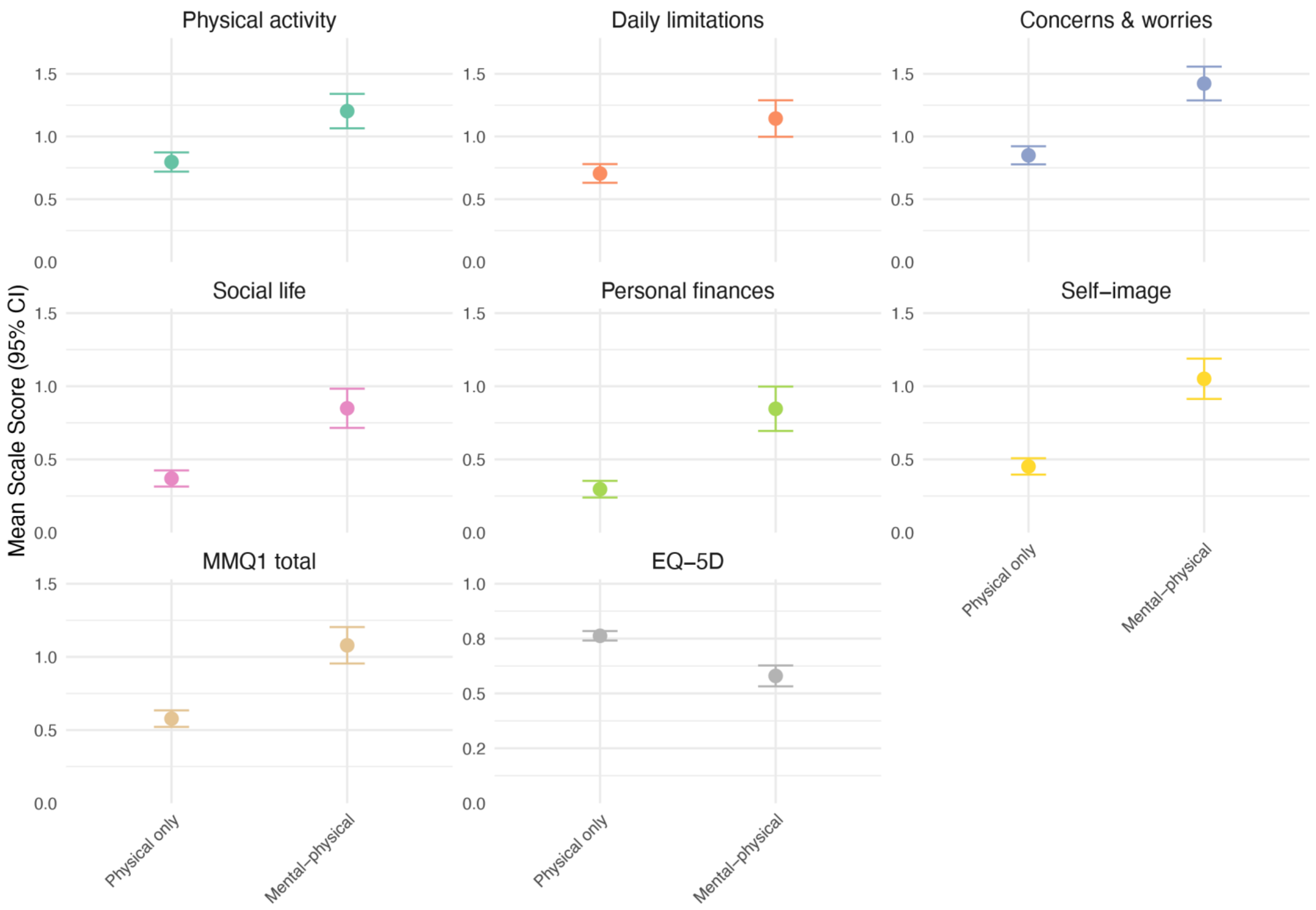

Supplementary

Note: Scale direction for EQ-5D-5L is opposite to that of MMQ1 and its subscales. Higher scores for MMQ1 indicate worse qulity of life, while higher scores on EQ-5D-5L indicate better quality of life. All MMQ scales have been standardised by diving mean score by number of items.

Figure 3. Variation in mean outcome scores according to global self-rated quality of life

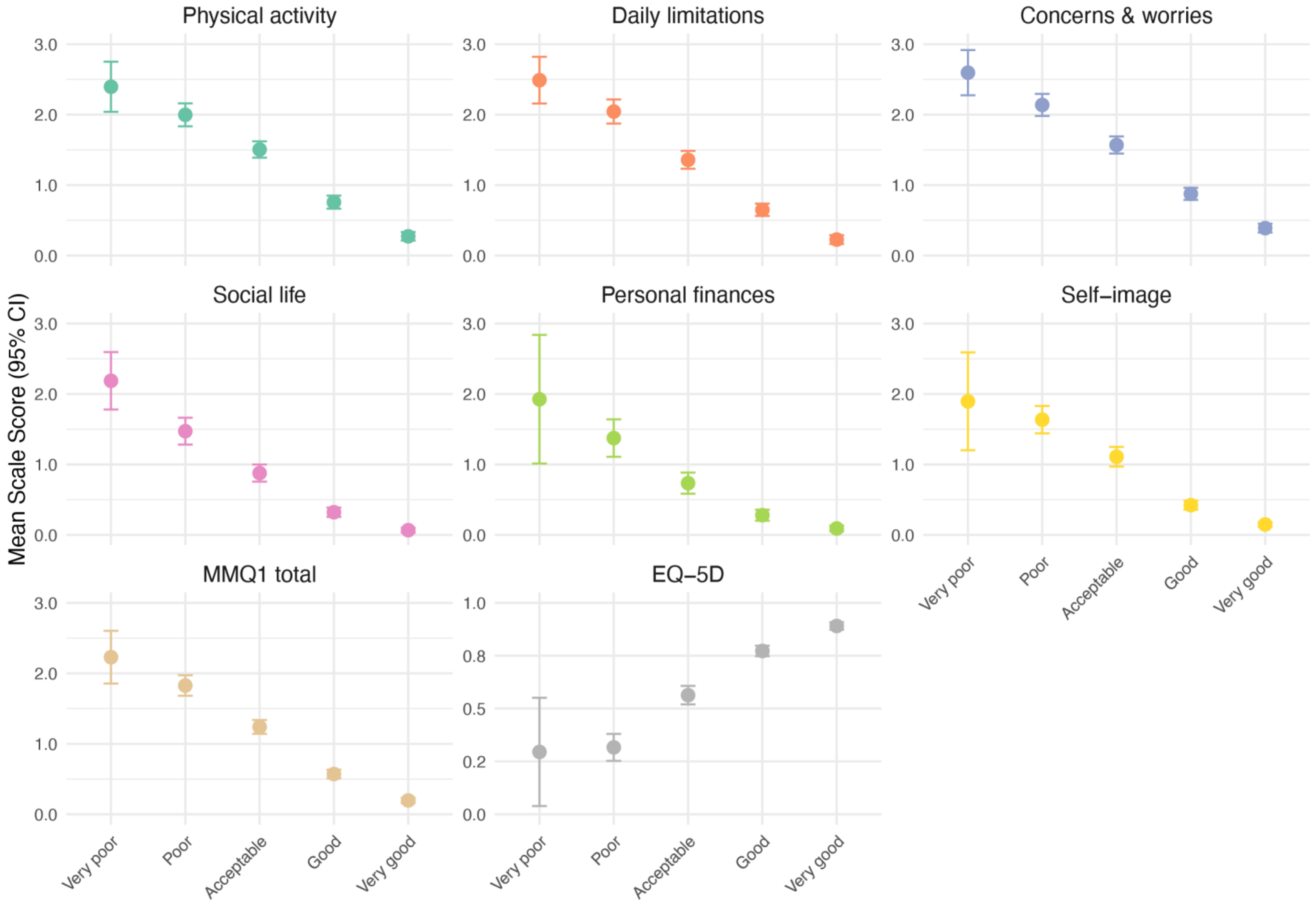

Supplementary

Note: Scale direction for EQ-5D-5L is opposite to that of MMQ1 and its subscales. Higher scores for MMQ1 indicate worse qulity of life, while higher scores on EQ-5D-5L indicate better quality of life. All MMQ scales have been standardised by diving mean score by number of items.

Figure 4. Variation in mean outcome scores according to age group

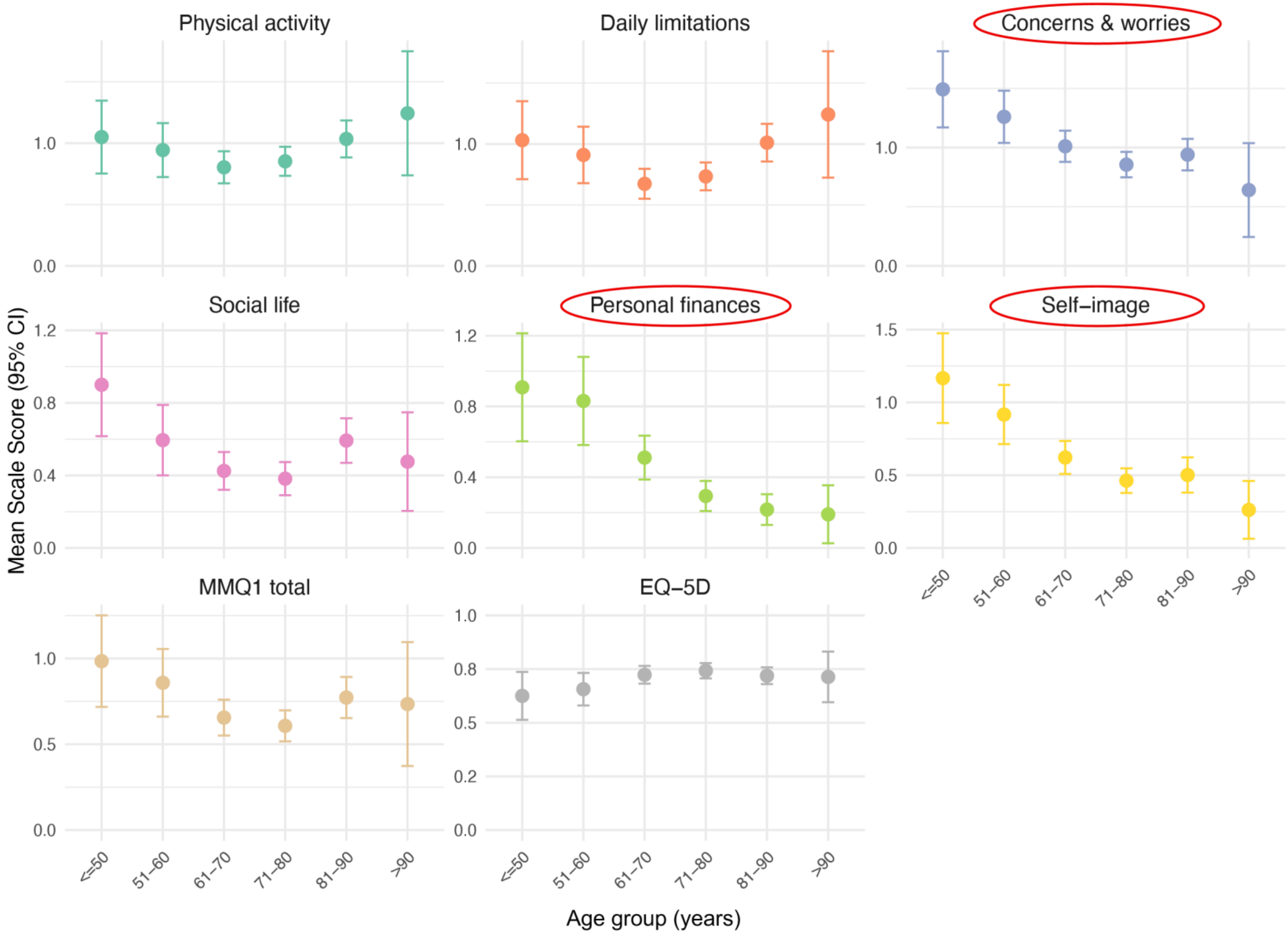

Supplementary

Note: Scale direction for EQ-5D-5L is opposite to that of MMQ1 and its subscales. Higher scores for MMQ1 indicate worse qulity of life, while higher scores on EQ-5D-5L indicate better quality of life. All MMQ scales have been standardised by diving mean score by number of items. Red circles highlight scales with a significant (p <0.01) association with age on the regression analyses.

Figure 5. One-vs-One Receiver Operating Characteristic (ROC) curves for each scale.

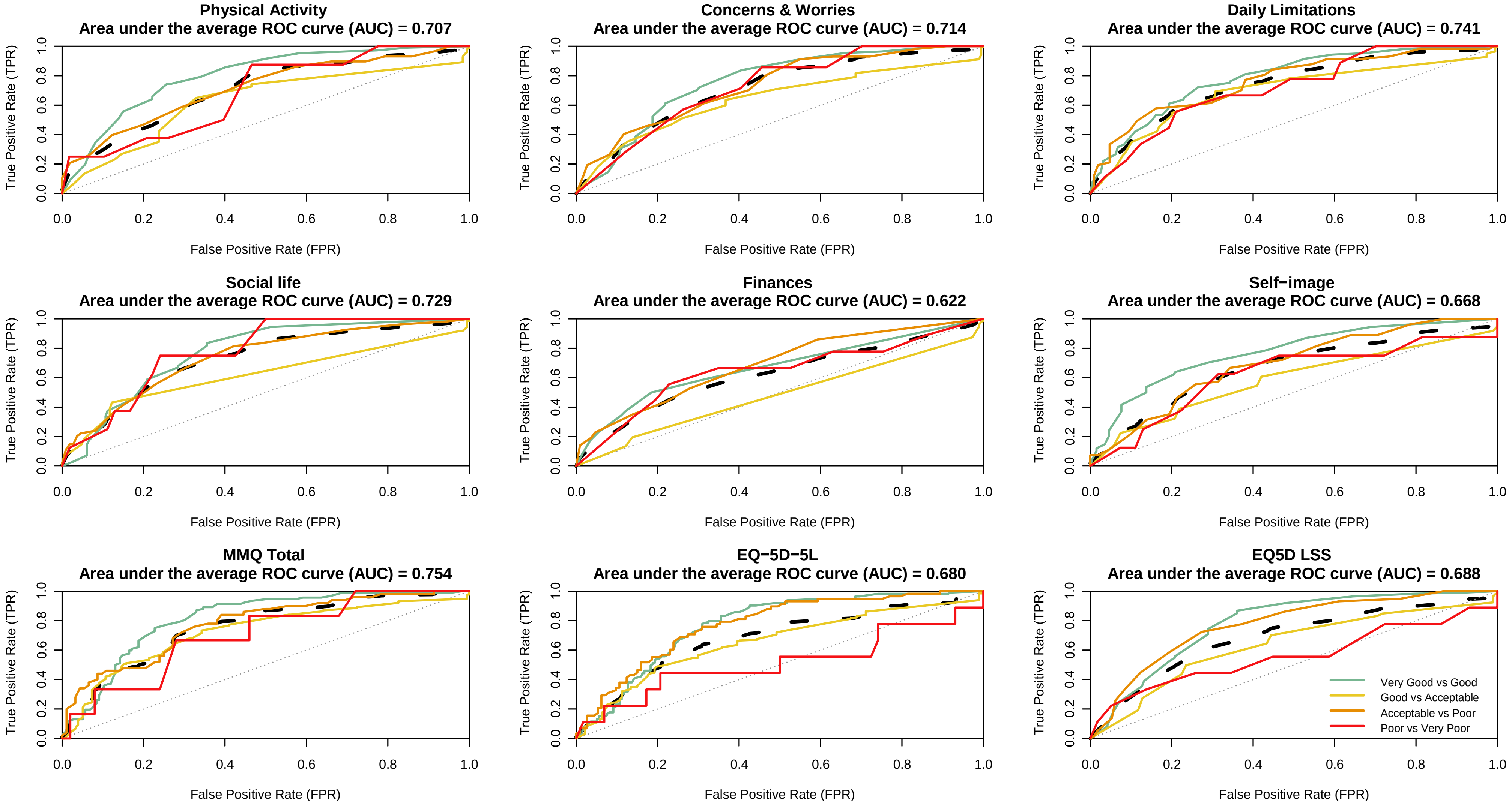

Supplementary

Each mini-plot represents a separate scale: six MMQ1 domains, MMQ1 Total, EQ-5D-5L utilitiy score, and EQ-5D-5L level sum score (LSS).

Each coloured line represents a pairwise class comparison (e.g., Poor vs Very Poor = red line) plotting the true positive rate against the false positive rate, showing how well the scale distinguishes between these classes. The thick black dashed line represents the average performance across all classes. The straight diagonal dotted line represents random change (AUC = 0.5)

Supplementary Table 1. Comparison of completers and non-completers

P-values for comparison between completers and non-completers for long-term condition (LTC) count, presence of mental-physical multimorbidity (MPM), deprivation, sex, age (≤65 vs 65+).

		Completers (n)	Noncompleters (n)	P value
Age (years)				0.57
	<65	152	51	
	65+	359	60	
Sex				0.06
	Male	259	51	
	Female	254	31	
Deprivation group				0.96
	High	143	24	
	Mixed	300	47	
	Low	71	12	
Self-reported LTC count				0.19
	0-1	52	7	
	2-3	210	26	
	4-6	207	42	
	6+	85	20	
Multimorbidity type				0.80
	Mental-physical	140	25	
	Physical only	322	7	
	Missing	52	51	

LTC = Long-term condition
